# Documenting legal status: a systematic review of measurement of undocumented status in health research

**DOI:** 10.1186/s40985-017-0073-4

**Published:** 2017-11-29

**Authors:** Maria-Elena De Trinidad Young, Daniel S. Madrigal

**Affiliations:** 1 Department of Community Health Sciences, Fielding School of Public Health, University of California, Los Angeles, 650 Charles E. Young Drive South, 36-071 CHS, Box 951772, Los Angeles, CA 90095-1772 USA; 2 California Environmental Health Tracking Program, Public Health Institute, 555 12th Street, 10th Floor, Oakland, CA 94607 USA

**Keywords:** Undocumented status, Measurement, Research methods, Systematic review

## Abstract

**Background:**

Undocumented status is rarely measured in health research, yet it influences the lives and well-being of immigrants. The growing body of research on undocumented status and health shows the need to assess the measurement of this legal status. We discuss the definition of undocumented status, conduct a systematic review of the methodological approaches currently taken to measure undocumented status of immigrants in the USA, and discuss recommendations for advancement of measurement methods.

**Methods:**

We conducted a systematic review of 61 studies indexed in PubMed, conducted in the USA, and published from 2004 to 2014. We categorized each of the studies’ data source and type, measurement type, and information for classifying undocumented participants. Studies used self-reported or proxy measures of legal status.

**Results:**

Information to classify undocumented participants included self-reported status, possession of a Social Security number, possession of health insurance or institutional resources, concern about deportation, and participant characteristics. Findings show it is feasible to collect self-reported measures of undocumented status.

**Conclusions:**

We recommend that researchers collect self-reported measures of undocumented status whenever possible and limit the use of proxy measures. Validated and standardized measures are needed for within and across country measurement. Authors should provide methodological information about measurement in publications. Finally, individuals who are undocumented should be included in the development of these methodologies.

**Trial registration:**

This systematic review is not registered.

## Background

Undocumented status is rarely measured in health research, yet it influences the lives and well-being of immigrants [1]. Data on immigrants’ legal status is sensitive, and its collection poses risks to research participants. A breach of privacy or confidentiality could result in disclosure of undocumented status and harmful legal repercussions for participants. Asking about legal status in the research setting may create discomfort, damage trust, and, overall, produce a “chilling effect” among participants [1–3]. As a result, most representative health and population surveys, such as the Current Population Survey or National Health Interview Survey in the USA, only ask participants’ country of origin and citizenship [1, 6]. Researchers who have examined the health impact of undocumented status in the USA, therefore, have relied on regional population health surveys that include questions about legal status, such as the Los Angeles Family and Neighborhood Survey or California Health Interview Survey, or have developed their own community-based surveys, conducted qualitative studies, and analyzed administrative data. This literature on undocumented status and health is growing, with reviews on the topic [7, 8] and studies of undocumented status and health care access [9–11], mental health [12, 13], and chronic disease [14, 15]. Given the increasingly hostile environment towards undocumented immigrants globally, this area of research has the ability to influence health policy and advance health equity for immigrant populations at the same time that thoughtful, ethical, and rigorous approaches are needed [16]. Yet, the lack of data on undocumented status continues to hinder the advancement of knowledge about the health of the undocumented population and the health impact of legal status.

This growing body of literature shows the need to understand and assess the methods for measuring undocumented status. Currently, recommendations about research with undocumented populations tend to focus on cautions of when to not measure legal status and there is limited methodological guidance of how to measure it in an ethically sound manner [4, 5]. Across existing studies and methodologies, no standardized measure exists to identify the undocumented status of participants. To date, there has been no examination of the approaches used to measure undocumented status in health research, although a recent study examined item response on surveys that ask about legal status [1]. Improved measurement of undocumented status will not only improve research methodology but will advance the principles of public health and other health research disciplines to address the fundamental causes of disease and respect the experiences of communities [17]. Given the risks involved in asking research participants about their legal status, an assessment of different approaches is critical to inform researchers in their selection of measures and methods. An assessment of existing measures of undocumented status can also inform the development of rigorous measurement methods. Therefore, in this paper, we examine the approaches currently used in health research to measure the undocumented status of immigrants in the USA, where a range of methodologies, such as population surveys and ethnographic studies, have been used to study undocumented populations. We discuss the definition of undocumented status, conduct a systematic review of the methodological approaches currently taken to measure undocumented status, and discuss recommendations for advancement of measurement methods.

### What is undocumented status?

While the terms undocumented, unauthorized, or illegal are widely used in academic and popular discourse, they refer to a category that is not as clear as generally assumed. The specific legal position of those who are undocumented varies from country to country because of distinct immigration laws. Regardless of the specific national context, undocumented status is one of many positions within the “axis of stratification” of a nation’s hierarchy of citizenship ([18], p. 1006). Legal scholar Linda Bosniak describes citizenship as a position of “formal legal standing” and “entitlement to, and enjoyment of, rights” that is defined by actual (e.g., legal) and symbolic (e.g., social) boundaries of inclusion or exclusion [19]. Such boundaries have real-world implications for individuals’ social position and rights [20]. Similar to citizenship, undocumented status can be defined by identifying its legal and social boundaries and the implications that those boundaries have for the lives of immigrants. To establish a definition of undocumented status in health research, we describe its legal and social elements within the hierarchy of citizenship in the USA, where it was estimated that in 2015 that 11.2 were undocumented [21].

A central legal element of undocumented status is the US federal immigration law that creates the boundaries of each legal status. The federal government has sole power to determine who can or cannot officially enter the country, determining who will be granted a lawful status. Legal statuses include naturalized citizenship or Lawful Permanent Residency—often referred to as “documented statuses”—and temporary statuses—often referred to as “twilight statuses” [22]. The lack of one of these statuses is referred to as “undocumented status.” Undocumented status, however, is a derivative, not statutorily an established status. Legal scholar Hiroshi Motomura asserts that it rests within a unique place “outside the law” [23]. The Immigration and Nationality Act of 1965—the body of Federal code that establishes current US immigration law—does not include “undocumented status” as an immigration category [24]. The act does, however, outline the penalties for “illegal entrants,” “immigration violators,” and “aliens unlawfully present”—the consequences for possessing a position not intended to legally exist [25].

Federal, state, and local policies together form additional legal elements of undocumented status. These levels of government possess varying authority to establish the rights that correspond to each legal status group. Through federal laws and policies, those who are undocumented are excluded from authorized employment, most public benefits, and other social and economic resources [23]. These individuals do receive some constitutional protections, for example, the US Supreme Court decision *Plyer v. Doe*, 457 US 202 (1982) established that undocumented children have a right to public primary and secondary education. Similarly, state and local laws can expand or restrict the rights of undocumented immigrants in areas such as health care, education, employment, or driver’s licenses [26, 27]. These legal boundaries define the significance of undocumented status in relation to the full rights of citizenship.

While legal elements of undocumented status are central in shaping its position in the citizenship hierarchy, the significance of being undocumented is not inherent to its position of formal legal exclusion. Rather, the implications of being undocumented are produced by social forces that create and reinforce this subordinate position in the nation’s citizenship hierarchy [28]. Its social elements further define the boundaries of undocumented status by determining lines of inclusion or exclusion within the society and social constructions of “citizenship” [29, 30]. For example, undocumented status may result in stigma for some individuals as a result of social attitudes or practices in their workplace or school [31]. This can, in turn, shape the circumstances and conditions under which undocumented immigrants are able to socially, economically, and politically integrate into US society [32, 33]. In addition, these social elements have shifted over time with changes in attitudes towards immigrants [34]. Social and legal elements can directly influence or reinforce one another. For example, during periods of greater xenophobia or political polarization, states and localities may pass more restrictive immigration policies [35]. Legal and social elements work to give significance to undocumented status and, critical for health research, produce the consequences of this status for the lives of immigrants.

To understand and assess current approaches to measuring undocumented status in health research, we conducted a systematic review of health literature on immigrant populations in the USA that examined the type and sources of data collected, the type of measurement instruments used to measure undocumented status, and the information used to classify those who are undocumented.

## Methods

**Fig. 1:**
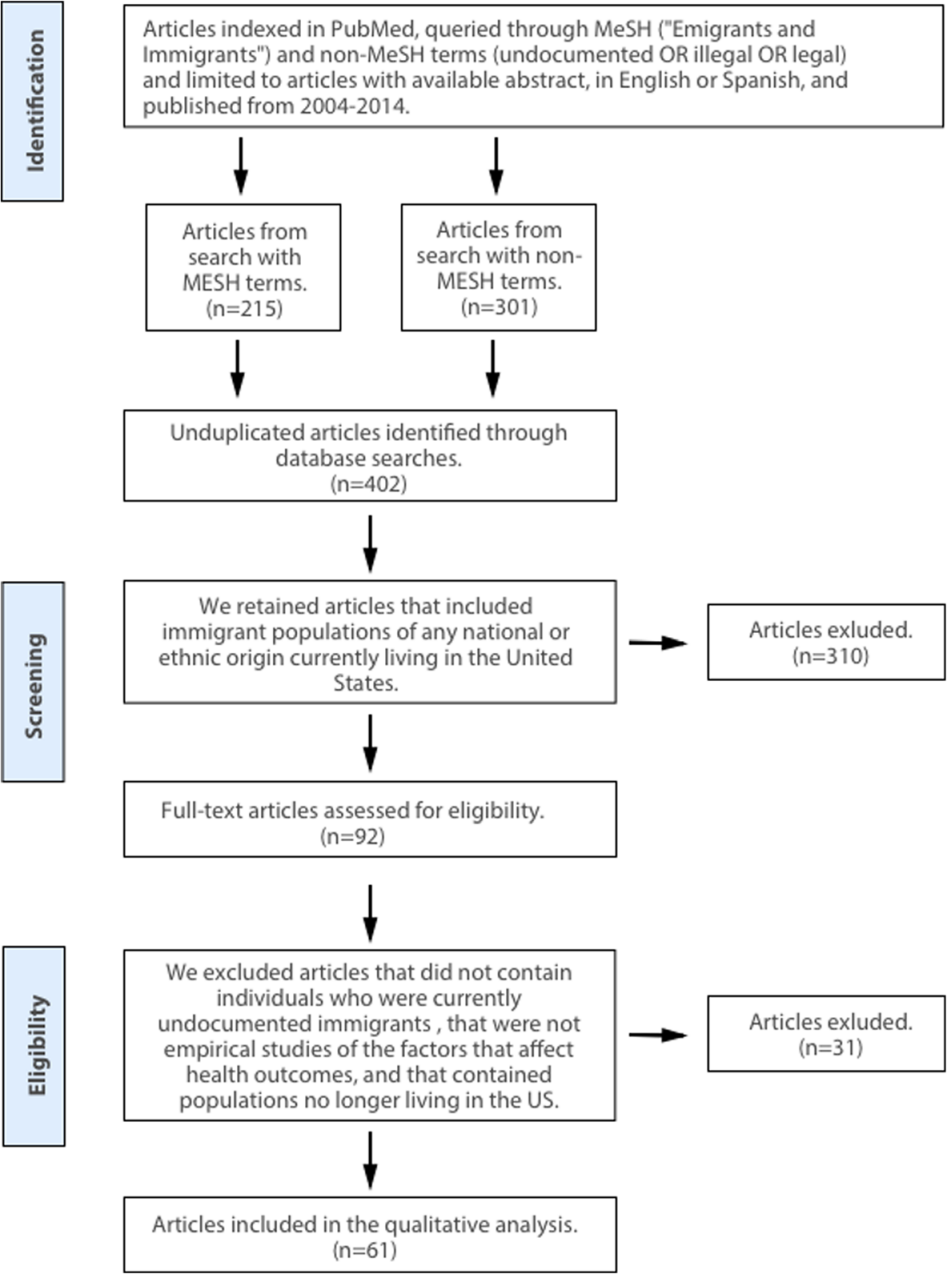
PRISMA diagram of literature review: identification, screening, and eligibility of reviewed articles

This systematic review was conducted according to the Preferred Reporting Items for Systematic Reviews and Meta-Analyses (PRISMA) guidelines [36]. Figure 1 presents the process by which we identified, screened, and selected eligible articles to obtain a sample of recent health studies that included measures of undocumented status.

### Article identification, screening, and eligibility

We queried articles indexed in PubMed to identify studies that included undocumented populations. This database contains a broad collection of health research articles maintained by the National Library of Medicine, as well as peer-reviewed articles from studies funded by the National Institutes of Health and other major studies that influence research and practice in the field. We included articles published in the 10 years preceding the beginning of the review, from 2004 to 2014, to produce a sample of studies that represent and influence contemporary research on legal status and health.

We used the following combinations of MeSH and non-MeSH terms: “Emigrants and Immigrants” [Mesh] and [undocumented OR illegal OR legal] and applied filters to limit the sample to articles with an available abstract and published in English or Spanish (Fig. 1). This produced a final, non-duplicated sample of 402 articles which we each independently reviewed. The inclusion criteria were designed to identify the research articles that included research on undocumented populations and in which we could examine the assumptions and methods guiding measurement of undocumented status. First, we retained articles that included research studies on immigrant populations of any national or ethnic origin in the USA, for a total of 92. Articles from immigrant populations outside of the USA were excluded, as the legal and social elements of undocumented status vary across countries. We then each independently reviewed the abstracts of the 92 articles and, when necessary, reviewed the full text. We further excluded those that did not contain individuals who were currently undocumented immigrants (e.g., only immigrants with lawful permanent status) that were not empirical studies (e.g., theoretical or policy papers) of the factors that affect health outcomes (e.g., physical or mental health, health care access) and that contained populations no longer living in the USA (e.g. individuals who have been deported). This resulted in the final sample of 61 articles with empirical research that included undocumented individuals living in the USA at the time of the study (Table 1).

**Table 1:** Included studies by data source, data type, measure type, and information used to measure undocumented status

Author(s), year	Title	Study size (*n*)	Study population	Data type^c^	Measure type^d^	Piece of information used
Direct data sources^a^						
Bacallao and Smokoski, 2009	Entre dos mundos/between two worlds: bicultural development in context.	26	Mexican adolescents and their parents	Qualitative—interviews	Unknown	Unknown
Bacallao and Smokoski, 2013	Obstacles to getting ahead: how assimilation mechanisms impact undocumented Mexican immigrant families.	10	Undocumented Mexican immigrant families	Qualitative—interviews	Unknown	Unknown
Brabeck and Guzman, 2009	Exploring Mexican-origin intimate partner abuse survivors’ help-seeking within their sociocultural contexts.	75	Mexican immigrant and Mexican-American women survivors of domestic violence	Survey—investigator-initiated	Unknown	Unknown
Campesino et al., 2009	Counternarratives of Mexican-origin women with breast cancer	10	Monolingual Spanish-speaking immigrants receiving breast cancer treatment	Qualitative—interviews	Self-reported	Self-reported undocumented status
Cartwright, 2011	Immigrant dreams: legal pathologies and structural vulnerabilities along the immigration continuum.	196	Mexican immigrants	Qualitative—ethnography	Unknown	Unknown
Cavazos-Rehg et al., 2007	Legal status, emotional well-being and subjective health status of Latino immigrants.	143	Latino immigrants	Survey—investigator-initiated	Proxy	Concern about deportation
Chandler et al., 2012	No me póngan mucha importancia: care-seeking experiences of undocumented Mexican immigrant women with chronic illness.	26	Undocumented Mexican immigrant women	Qualitative—ethnography, interviews	Unknown	Unknown
Chavez, 2012	Undocumented immigrants and their use of medical services in Orange County, California.	1201	Residents of Orange County, CA	Survey—investigator-initiated	Self-reported	Self-reported legal status
Chen, 2009	Predictors of breast examination practices of Chinese immigrants.	135	Chinese immigrant women	Survey—investigator-initiated	Self-reported	Unknown
Chu et al., 2003	Effects of post-migration factors on PTSD outcomes among immigrant survivors of political violence.	875	Immigrant survivors of political violence	Clinical—clinical intake interviews	Self-reported	Unknown
Cleaveland, 2010	We are not criminals: “social work advocacy and unauthorized migrants.”	32	Latino day laborers	Qualitative—ethnography, interviews	Unknown	Unknown
Coffman et al., 2009	Self-prescription practices in recent Latino immigrants.	19	Latino immigrants	Qualitative—focus groups	Unknown	Unknown
Dang et al., 2012	Sociocultural and structural barriers to care among undocumented Latino immigrants with HIV infection.	22	Undocumented Latino immigrants	Qualitative—interviews	Unknown	Unknown
Deb-Sossa et al., 2013	Experiences of undocumented Mexican migrant women when accessing sexual and reproductive health services in California, USA: a case study	8	Undocumented Mexican immigrant women	Qualitative—interviews	Self-reported	Unknown
Dillon et al., 2013	Acculturative stress and diminishing family cohesion among recent Latino immigrants.	405	Cuban, Colombian, Honduran, Nicaraguan, and Venezuelan immigrants	Survey—investigator-initiated	Self-reported	Self-reported undocumented status
Flores et al., 2006	Why are Latinos the most uninsured racial/ethnic group of US children? A community-based study of risk factors for and consequences of being an uninsured Latino child.	1100	Latino parents	Survey—investigator-initiated	Self-reported	Unknown
Fuentes-Afflick and Hessol, 2009	Immigration status and use of health services among Latina women in the San Francisco Bay Area.	710	Latina women mothers who recently gave birth	Survey—investigator-initiated	Self-reported	Self-reported undocumented status
Fuentes-Afflick et al., 2006	Use of prenatal care by Hispanic women after welfare reform.	3957	Latina women mothers who recently gave birth	Survey—investigator-initiated	Self-reported	Self-reported undocumented status
Goldman, 2005	Legal status and health insurance among immigrants.	1056	Residents of Los Angeles County, CA	Survey—Los Angeles Family and Neighborhood Survey	Self-reported	Self-reported legal status
Guendelman et al., 2005	Overcoming the odds: access to care for immigrant children in working poor families in California.	4440	Children and adolescents	Survey—California Health Interview Survey	Self-reported	Unknown
Guh et al., 2011	Missed opportunities to prevent tuberculosis in foreign-born persons, Connecticut, 2005–2008.	346	Immigrants with suspected TB	Clinical—surveillance data	Self-reported	Unknown
Hadley et al., 2008	Hunger and health among undocumented Mexican migrants in a US urban area.	430	Mexican immigrants	Survey—investigator-initiated	Self-reported	Self-reported legal status
Heyman et al., 2009	Healthcare access and barriers for unauthorized immigrants in El Paso County, Texas.	52	Undocumented immigrants	Unknown—interviews	Unknown	Unknown
Holmes and Marcelli, 2012	Neighborhoods and systemic inflammation: high CRP among legal and unauthorized Brazilian migrants.	307	Brazilian immigrants	Survey—ethnography, interviews	Self-reported	Unknown
Holmes, 2006	An ethnographic study of the social context of migrant health in the United States.	n/a	Migrant farm workers	Qualitative—investigator-initiated	Unknown	Unknown
Ingram et al., 2010	Experiences of immigrant women who self-petition under the Violence Against Women Act.	21	Immigrant women who filed VAWA self-petitions	Qualitative—interviews, focus groups	Unknown	Unknown
Loue et al., 2005	Welfare and immigration reform and use of prenatal care among women of Mexican ethnicity in San Diego, California.	157	Mexican immigrant and Mexican-American women	Qualitative—interviews	Unknown	Unknown
Maldonado et al., 2013	Fear of discovery among Latino immigrants presenting to the emergency department.	1007	Undocumented Latino immigrants	Survey—investigator-initiated	Self-reported	Self-reported legal status
Marín et al., 2009	Evidence of organizational injustice in poultry processing plants: possible effects on occupational health and safety among Latino workers in North Carolina.	200	Poultry workers	Survey—investigator initiated	Unknown	Unknown
Marshall et al., 2005	Health status and access to health care of documented and undocumented immigrant Latino women.	197	Latina immigrants	Survey—investigator-initiated	Self-reported	Self-reported legal status
Momper et al., 2009	The prevalence and types of gambling among undocumented Mexican immigrants in New York City.	431	Undocumented Mexicans	Survey—investigator-initiated	Unknown	Unknown
Montealegre et al., 2005	HIV testing behaviors among undocumented Central American immigrant women in Houston, Texas.	182	Undocumented Central American immigrant women	Unknown—investigator-initiated	Unknown	Unknown
Montealegre et al., 2012	Prevalence of HIV risk behaviors among undocumented Central American immigrant women in Houston, Texas.	210	Central American immigrants	Survey—investigator-initiated	Self-report	Unknown
Morano et al., 2013	Latent tuberculosis infection: screening and treatment in an urban setting.	357	TB patients	Clinical—baseline clinical interview	Unknown	Unknown
Nandi et al., 2008	Access to and use of health services among undocumented Mexican immigrants in a US urban area.	431	Undocumented Mexican immigrants	Survey—investigator-initiated	Self-reported	Unknown
Negi, 2013	Battling discrimination and social isolation: psychological distress among Latino day laborers.	150	Latino day laborers	Qualitative—investigator-initiated	Proxy	Personal or population characteristic
Ordoñez, 2012	Boots for my Sancho’: structural vulnerability among Latin American day labourers in Berkeley, California.	10	Latino day laborers	Qualitative—ethnography	Unknown	Unknown
Ornelas et al., 2013	Perceived barriers to opportunity and their relation to substance use among Latino immigrant men.	291	Latino immigrant males	Survey—investigator-initiated	Self-reported	Unknown
Ortega et al., 2007	Health care access, use of services, and experiences among undocumented Mexicans and other Latinos.	42,004	Adults in California	Survey—California Health Interview Survey	Self-reported	Self-reported legal status
Pivnick et al., 2010	Accessing primary care: HIV+ Caribbean immigrants in the Bronx.	55	HIV-positive Caribbean immigrants	Survey—ethnography, interviews	Self-reported	Self-reported undocumented status
Potochnik et al., 2010	Depression and anxiety among first-generation immigrant Latino youth: key correlates and implications for future research.	281	First-generation Latino immigrant youth	Survey—investigator-initiated	Self-reported	Unknown
Prentice et al., 2005	Immigration status and health insurance coverage: who gains? Who loses?	2130	Residents of Los Angeles County, CA	Survey—Los Angeles Family and Neighborhood Survey	Self-reported	Self-reported legal status
Standish et al., 2010	Household density among undocumented Mexican immigrants in New York City.	404	Undocumented Mexican immigrants	Survey—investigator-initiated	Self-reported	Unknown
Stevens et al., 2010	Health insurance and access to care for families with young children in California, 2001–2005: differences by immigration status.	37,236	Families with children in California	Survey—California Health Interview Survey	Unknown	Unknown
Valdez et al., 2013	Why we stay: “immigrants’ motivations for remaining in communities impacted by anti-immigration policy.”	25	Mexican immigrant parents	Qualitative—focus groups	Proxy	Personal or population characteristic
Vargas-Bustamante et al., 2012	Variations in healthcare access and utilization among Mexican immigrants: the role of documentation status.	51,048	Representative sample of CA	Survey—California Health Interview Survey	Self-reported	Self-reported legal status
Walter et al., 2004	Masculinity and undocumented labor migration: injured Latino day laborers in San Francisco.	40	Day laborers	Qualitative—ethnography	Unknown	Unknown
Yoshikawa et al., 2008	Access to institutional resources as a measure of social exclusion: relations with family process and cognitive development in the context of immigration.	181	Dominican, Mexican, and Black mothers of 24-month-old children	Survey—investigator-initiated	Proxy	Possession of institutional resources
Indirect data sources^b^						
Achkar et al., 2008	Differences in clinical presentation among persons with pulmonary tuberculosis: a comparison of documented and undocumented foreign-born versus US-born persons.	194	TB patients	Clinical—medical records	Proxy	Unknown
Appleby et al., 2008	The impact of immigration on psychiatric hospitalization in Illinois from 1993 to 2003.	13,408	Individuals admitted to public psychiatric hospitals	Clinical—state hospital Clinical Information System	Proxy	Social Security number
Dubard and Massing, 2007	Trends in Emergency Medicaid expenditures for recent and undocumented immigrants.	48,391	Immigrants receiving Emergency Medicaid	Administrative—state Medicaid data	Proxy	Type of health insurance
Hacker et al., 2011	The impact of Immigration and Customs Enforcement on immigrant health: perceptions of immigrants in Everett, Massachusetts, USA.	52	Immigrants	Qualitative—focus groups	Unknown	Unknown
Korinek et al., 2011	Prenatal care among immigrant and racial-ethnic minority women in a new immigrant destination: exploring the impact of immigrant legal status.	300,000+	Women who gave birth in Utah	Administrative—Utah population database	Proxy	Possession of institutional resources
Leclere et al., 2012	The jornalero: perceptions of health care resources of immigrant day laborers.	20	Day laborers	Qualitative—interviews	Proxy	Personal or population characteristic
Linden et al., 2012	Kidney transplantation in undocumented immigrants with ESRD: a policy whose time has come?	132	ESRD immigrant patients	Clinical—patient survey	Proxy	Social Security number
Lowry et al., 2010	Possibilities and challenges in occupational injury surveillance of day laborers.	160	Day laborers	Clinical—hospital trauma registry	Proxy	Social Security number
Mitchell et al., 2012	Who will cover the cost of undocumented immigrant trauma care?	36,525	Patients diagnosed with trauma	Clinical-quantitative —data extraction (e.g., insurance files)	Proxy	Type of health insurance
Poon et al., 2013	Treatment outcomes in undocumented Hispanic immigrants with HIV infection	1620	HIV-positive Hispanic immigrants	Clinical—patient survey	Proxy	Social Security number
Rasmussen et al., 2013	The subjective experience of trauma and subsequent PTSD in a sample of undocumented immigrants.	212	Individuals presenting at US ports of entry	Qualitative—interviews	Proxy	Personal or population characteristic
Reed et al., 2005	Birth outcomes in Colorado’s undocumented immigrant population	5961	Women who gave birth in Colorado	Administrative—linked Medicaid and birth certificate data	Proxy	Type of health insurance
Stimpson et al., 2013	Unauthorized immigrants spend less than other immigrants and US natives on health care.	NA	Medical care expenditures	Survey—Medical Expenditure Panel Survey	Self-reported	Statistical modeling

^a^Direct data sources are those collected directly from participants for the purpose of classifying their legal status (*n* = 48)

^b^Indirect data sources are those collected for research or data purposes other than classifying individuals’ legal status (*n* = 13)

^c^Survey data is collected through structured survey methods (*n* = 28). Qualitative data is collected through semi- or unstructured qualitative methods (*n* = 18). Administrative data is collected from governmental records (*n* = 4). Clinical data is collected for purpose of providing health care (*n* = 9). Those marked unknown could not be determined (*n* = 2)

^d^Self-reported are those in which participants provided explicit information related to their legal status (*n* = 26). Proxy measures are those in which data were used to derive an approximation of participants’ legal status (*n* = 15). Those marked unknown could not be determined (*n* = 20)

### Categorization of measurement approach

For each paper, we extracted the text that described the methodology used to measure undocumented status and documented the study size, population, and description of data source (Table 1). Each author independently reviewed the text of each article and developed initial categories to describe the studies’ measurement process. We developed four domains that categorize all of the studies according to the common approaches used to measure undocumented status and coded each according to data source, data type, measurement type, and information used to classify undocumented status (Table 1). Because many studies provided incomplete information about their methods, we incorporated non-report into the coding scheme to document the extent to which methodological information is reported and made available to other researchers.

The first domain was the data source used in the study and from which measurement was conducted. Articles were coded as “direct” if data were collected from participants by researchers for the purpose of classifying their legal status. This included studies that analyzed secondary data sets that had been originally collected from research participants. Articles were coded “indirect” if the data that was used for measurement were not collected for the purpose of classifying individuals’ legal status but for other research or data collection purposes.

The second domain was the type of data that were collected. Studies were coded as using qualitative data if authors collected data through unstructured or semi-structured qualitative methods, survey data if authors collected quantitative data with structured instruments, administrative data if authors collected governmental records (e.g., Department of Motor Vehicles), or clinical data if authors collected quantitative or qualitative data generated by health care institutions for the purpose of providing health care.

The third domain was the type of measure that was applied during data collection or generated from the collected data. Articles were coded as using a self-reported measure if participants provided explicit information related to their legal status. Articles were coded as creating a proxy measure if data on participant characteristics were used to derive an approximation of their undocumented status. Studies that did not report their measure type were coded “Unknown.”

The fourth domain was the information used to classify individuals’ undocumented status. Studies were coded as using one of the eight pieces of information: self-reported legal status with no explicit query about undocumented status, in which participants provided information in response to survey questions about other legal status categories, but not explicitly about whether or not they were undocumented; self-reported undocumented status, in which research participants provided explicit information about whether or not they were undocumented; possession of a Social Security number; type of health insurance; possession of institutional resources; statistical modeling; concern about deportation; or participants’ personal or population characteristics. Studies that did not report the piece of information used were coded “Unknown.”

We each independently applied the domain categories to the 61 articles. Where there were discrepancies in the two sets of codes, we reviewed the text and discussed the categories to determine which was the most appropriate.

## Results

Overall, 48 studies used direct and 13 used indirect data. Most of the studies (*n* = 26) used self-reported measures, while 15 used proxy measures. The measure type was unknown for the 20 studies. The majority of studies with self-reported measures used direct data sources that had survey or qualitative data. For example, among studies using direct data, the majority (*n* = 28) used survey data from representative population surveys or from investigator-initiated surveys conducted among convenience samples. The remaining (*n* = 18) collected qualitative data through focus groups, interviews, life histories, and ethnographic participant-observation. Most of those with proxy measures used indirect data containing clinical and administrative data. The 13 studies that used indirect data obtained clinical data (*n* = 9) from hospital or health center records, including social worker records, a hospital trauma registry, and a state psychiatric hospital information system, or authors obtained administrative data (*n* = 4) from state insurance claim records, records of individuals seeking admission to the USA, or state driver license records. Five studies, however, used direct data to generate proxy measures. One of these collected survey data that inquired about whether or not participants were concerned about deportation and another inquired about whether or not individuals possessed a driver’s license or a bank account [37, 38].

Table 2 lists and describes the information used to classify undocumented status. It also includes the corresponding data source, data type, and measurement type for each, illustrating the process by which information on undocumented status was collected or generated. Thirty-two of the studies did not include sufficient detail in the description of their methods to be able to identify the specific piece of information used to classify undocumented status.

**Table 2:** Information that is used to classify undocumented status, by total number of studies, and corresponding data source, data type, and measure type

Information to classify undocumented status	*n*	Description	Source of data		Type of data				Type of measure	
			Direct	Indirect	Qualitative	Survey	Clinical	Administrative	Self-reported	Proxy
Self-reported legal status, with no explicit query about undocumented status	8	Participants reported whether or not they had another legal status, through survey questions, but were not explicitly asked whether or not they were undocumented	X			X			X	
Self-reported undocumented status	5	Participants reported explicitly whether or not they were undocumented either through survey questions or unprompted disclosure	X		X	X			X	
Possession of a Social Security number	4	Classified as undocumented if participants did not possess a Social Security number		X			X			X
Type of health insurance	3	Classified as undocumented if participants possessed Emergency Medicaid or lacked insurance (e.g., “self-pay”)		X			X	X		X
Possession of institutional resources	2	Classified as undocumented if participant lacked institutional resources, such as a driver’s license or bank account	X	X		X		X		X
Statistical modeling	1	Classified as undocumented from statistical prediction models of demographic and economic characteristics		X		X				X
Concern about deportation	1	Classified as undocumented if participants expressed concern about deportation, such as “I have thought that if I went to a social or government agency I would be deported”	X			X				X
Personal or population characteristic	4	Classified as undocumented if participants belonged to a specific group, such as a day laborer, a parent in high-immigrant enrollment schools, or a person attempting to enter at port of entry	X	X	X			X		X
Unknown	33		X		X	X	X		X	
Total			48	13	18	28	9	4	26	15

### Information from self-reported measures

All of the self-reported measures yielded information about some aspect of participants’ legal status; only five studies, however, collected explicit self-reported information about whether or not a participant was undocumented. Eleven studies classified undocumented status through survey data that included sequential, deductive questions about legal status—beginning with whether or not a participant was a US citizen followed by various lawful statuses. In three of these studies, the surveys ended with an explicit question about undocumented status. In the remaining eight, the participants were not explicitly asked if they were undocumented, rather the questions were used to eliminate those respondents who reported possessing a lawful status (e.g., US citizen, Lawful Permanent Resident)—which indicated that they are not undocumented. The remaining were then classified as undocumented.

There was variation in the lawful status categories that were included in these survey questions. For example, one study asked two questions: “Are you a citizen of the United States?” And, if the response was no, it was followed by, “Are you a permanent resident with a green card?” Those who answered “no” were classified as undocumented [39]. Other surveys, including one with 14 different legal statuses, included categories of legal status such as asylum or refugee status, Temporary Protected Status, Permanent Residence under Color of Law (PRUCOL), parole, or a student or tourist visa [10, 40]. For example, one study described that, after determining that participants were not US born or Lawful Permanent Residents (LPRs), the authors “asked if they had been granted asylum, refugee status, temporary protected immigrant status, a student or tourist visa, or another document permitting them to stay in the US for a limited time. People answering affirmatively to any of these questions and reporting that their documents had not expired were classified as ‘nonimmigrant.’ The remainder of the foreign-born were classified as ‘undocumented’” [10].

The remaining two studies classified undocumented status through unprompted, self-disclosed information in qualitative data. The authors reported that they did not intend to collect information on undocumented status, but that all participants self-disclosed during open-ended interviews [9, 41].

### Information from proxy measures

Proxy measures included the following information to classify undocumented status: whether or not an individual had a Social Security number (SSN) (*n* = 4), type of health insurance that an individual possessed (*n* = 3), possession of institutional resources (*n* = 2), statistical modeling to predict undocumented status (*n* = 1), if participants reported concern about deportation (*n* = 1), and characteristics of the study sample (*n* = 4).

Generally, only immigrants who are legally present and authorized to work in the USA can possess a Social Security number, making this a proxy for whether or not a research participant is undocumented [42]. Studies that classified individuals based on possession of a SSN obtained this proxy measure from clinical data. For example, one study classified individuals as undocumented if they had no or an invalid SSN. The authors describe their criteria for identifying these individuals: “Invalid SSNs are series that have never been assigned by the US Social Security Administration. For the SSN “XXX-YY-ZZZZ,” invalid series included any combination containing XXX of 000 or 666, YY of 00, or ZZZZ of 0000. SSNs higher than 772-82-9999 were also invalid. ‘No SSN’ was defined as having a generically assigned 999-99-9999 series or no number in the [data set].” [43].

Most states in the USA exclude undocumented immigrants from access to resources [44], such as public health insurance or driver’s licenses. Therefore, in several studies, researchers used data on possession of these types of resources as a proxy for whether or not a participant was undocumented. Studies that classified individuals based on insurance type classified individuals as undocumented if they had received services using Emergency Medicaid and, in one study, if they were uninsured or “self-pay” patients and also had no SSN. For example, one study identified mothers in the state’s Medicaid records who had given birth under Emergency Medicaid, as “Emergency Medicaid is provided to undocumented non-citizens who are financially eligible for Medicaid. Emergency Medicaid only provides medical coverage for medical emergencies, which includes labor and delivery…Colorado Medicaid adds the letter ‘J’ to the identification number of all enrollees with Emergency Medicaid (EMJ). We obtained Medicaid records for all EMJ labor and delivery claims.” [45]. The institutional resources used to classify undocumented status included lack of a driver’s license, a driver privilege card, or a bank account. Specifically, “Household access to institutional resources was assessed through a 4-item index at the 14-month wave. Mothers were asked to indicate (yes/no) whether they or anyone in their household has (a) a checking account, (b) a savings account, (c) a credit card, and (d) a driver’s license. These items were then summed to create an index of household access to institutional resources” [38]. Concern about deportation was assessed through a single question, “I have thought that if I went to a social or government agency I would be deported” [37]. Predicted undocumented status was based on social and economic characteristics reported in the Medical Expenditure Panel Survey.

Finally, four articles used information about characteristics—presumed to be common to undocumented individuals—of the study population to estimate legal status. These were having the occupation of a day laborer, an individual seeking non-authorized admission at a US port of entry, and parents in title I elementary schools. In these studies, the authors used characteristics to define the study sample and, thus, intentionally avoid making a direct inquiry about individuals’ legal status.

In addition, five of the studies that used proxy measures applied the information criteria above exclusively to specific populations, generally Hispanics or recent immigrants. For example, in one study that used lacked of a SSN as a proxy for undocumented status, this criterion was applied solely to Hispanic participants [46]. In another study, lack of a SSN was combined with respondents’ “social history,” such as country of birth, time in the USA, and reason for migration to USA, to classify participants as undocumented [47].

## Discussion

We conducted a systematic review of the measurement of undocumented status in recent health research in the USA. Our findings show that researchers are engaging in this process across a variety of research contexts—from utilizing administrative data on driver’s licenses to engaging immigrants through ethnographic research. Despite the importance of each step in the measurement process, the majority of studies reviewed—33 of 61—did not provide complete information about their full process. For example, while 28 studies reported using survey data, only 14 of those studies specified what was asked of or reported by participants to be able to classify them as undocumented. Of 18 studies that used qualitative methods, only five provided complete information about each step. Given the elements of undocumented status and the complexity of individuals’ experience, these studies likely measure different experiences and definitions of undocumented status.

The studies reviewed here can be broadly described as using either a process to collect a self-reported measure or a process to derive a proxy measure. Our findings suggest that the use of either of these two approaches is determined by a researcher’s selection of a data source and type. Direct data collection allowed researchers to collect self-reported measures of undocumented status through surveys and qualitative methods. Among these self-reported measures, each used a different set of questions to collect information to classify individuals as undocumented. In only a small number of studies where participants asked directly or voluntarily disclosed that they were undocumented. In the remaining studies, questions about legal status categories were used to deductively determine which respondents were undocumented.

In contrast, use of indirect data required that researchers analyze available information to create proxy measures. Proxy measures were generally utilized where no existing self-reported measures of legal status existed in clinical or administrative data sets. However, in some studies, proxies were developed even when collecting direct data from research participants. In these studies, authors reported that they opted to not ask directly about legal status to avoid creating discomfort among participants [48]. Across all of the studies, we identified six unique pieces of information that served as proxy measures, from possession of a SSN to using statistical modeling to predict undocumented status based on socio-demographic characteristics.

### The feasibility of collecting self-reported measures of undocumented status

The studies reviewed here indicate that it is feasible to obtain self-reported information about individual’s legal status through both qualitative and survey methods. While most of the studies did not explicitly ask about whether or not a respondent was undocumented, five studies did obtain explicit information about undocumented status. All used similar methods as the other studies that asked about other categories of legal status, but that stopped short at explicit inquiry regarding undocumented status. This suggests that it is also feasible to explicitly inquire about undocumented status, a step in the measurement process that would provide more detailed measurement of legal status.

The approaches taken across these studies provide examples of strategies that can facilitate the collection of measures of undocumented status by building rapport with study participants. Given the sensitive nature of legal status and undocumented status, in particular, the authors’ employed approaches focused on preventing a “chilling effect” when directly inquiring about legal status. First, the collection of data can take place in a range of settings that allow participants to feel comfortable, including focus groups, interviews, participant observation, in-person surveys, and phone surveys. Second, in survey research settings, researchers can establish rapport prior to presenting legal status questions. For example, some studies described their process to obtain legal status information: “During the latter part of the interview, after the woman had developed familiarity with the interviewer and the interview process, we asked a series of questions about immigration status” [11] and, in another, “…[participants] filled out the questionnaire on their own, [researchers] read each question and response option aloud to the group” [49]. In both of these studies, the surveys included explicit questions about whether or not the respondent was undocumented. Qualitative research settings similarly provide a context for establishing rapport prior to inquiring about legal status. Indeed, in two of the qualitative studies in our review, the authors were able to obtain measures of undocumented status because participants self-disclosed without prompting. This suggests that given rapport with researchers, some participants are interested in and willing to discuss undocumented status in research.

These approaches correspond with the recommendations made by Massey and Capoferro [6] to combine survey and ethnographic methods, allowing for quantitative collection of information, but inclusion of legal status questions through less structured processes. Further, the feasibility of collecting and using self-reported legal status is supported by recent studies that have examined response rates to legal status questions in large representative, population surveys. Bachmeier and colleagues [1] found high response rates and little evidence of a “chilling effect.”

Notably, the authors of these studies did not explicitly report how they weighed the risks and benefits of measuring undocumented status, nor the measures taken to protect participant confidentiality [3]. While not explicitly mentioned, two approaches to protecting sensitive legal status information are to collect all data anonymously or to obtain a National Institutes of Health (NIH) Certificate of Confidentiality (CoC). By collecting data anonymously through one-time interviews or surveys, data on legal status is not linked to participant identifying information. When it is necessary to collect identifying information, such as for follow-up interviews, a CoC provides researchers with some protections against data disclosure [50]. Prentice et al. [51] highlight that CoCs provide researchers with protection against having to release some elements of their data. These can be obtained for any study, regardless of funding source, and the NIH now provides these automatically to all grant recipients.

### Proxy measures capture the social, not legal elements of undocumented status

The studies reviewed here indicate that proxy measures provide an alternative to self-reported measures when the data source does not include direct measures of legal status. In addition, proxy measures can be used when researchers determine that it is not feasible or safe to collect data directly from research participants. However, the information that serves as proxies is based on assumed social elements of undocumented status. Proxy measures, as a result, have significant limitations.

First, proxies may inadvertently reinforce stereotypes about the undocumented population and conflate one set of experiences with that of being undocumented. The four studies that sought to avoid asking about legal status applied population-level generalizations about undocumented immigrants to individual research respondents’ personal characteristics, such as being a day laborer or attempting unauthorized entry at a port of entry [12, 48, 52, 53]. As discussed above, the legal status is made up of both legal and social dimensions. The use of respondent characteristics as proxies relies on assumed social dimensions among the undocumented population, such as being in the low wage workforce or entering the USA on foot at the border. One limitation is that such characteristics do not apply to all undocumented immigrants. For example, not all day laborers are undocumented and some individuals seeking unauthorized entry are asylum seekers and are granted a lawful status. Second, the use of such characteristics may be counterproductive to efforts to understand and promote the well-being of the undocumented population by advancing overly narrow representations of the complex legal and social elements of this status. Specifically, while an individual’s personal characteristics may have been shaped by the experience of being undocumented, those characteristics are not the same as the legal dimensions that determine their legal status [54].

Second, proxies exclude some undocumented individuals and include some documented individuals. For example, the use of SSN, possession of a driver’s license, or type of health insurance as proxies are based in laws that establish identity and qualification for government services. They may, however, overlook that undocumented individuals may report having a SSN, either obtained fraudulently or during a period when they were documented or have obtained a license in a different state, and that some US-born individuals do not know or have access to identification or other institutional resources, such as those needed for voting [55]. Therefore, while these are certainly proxies for legal exclusions experienced by undocumented immigrants, they are also likely a proxy of social or economic marginalization, independent of citizenship or legal status.

### Lack of validity and reliability of measures of undocumented status

The numerous approaches to measuring undocumented status raise the question: What is being measured? And how well? No studies included here reported on the validity and reliability of the measures used, response rates, or handling of missing data (e.g., imputation of legal status). Beyond that, each stage in the measurement process—implicitly or explicitly—determines which elements of undocumented status are being measured. First, the data source determines who within the nation’s very heterogeneous immigrant and undocumented populations are included and measured. Although undocumented individuals can be found among immigrants of different national origins, the majority of studies that we identified focused on immigrants from Latin America (see Table 1). In addition, in some of the studies, the criteria for classification were only applied to specific populations. For example, in one study that used possession of driver’s license to classify undocumented individuals, the additional following criteria were applied: “mother’s nativity, or country of birth (whether the United States, or elsewhere), and whether or not the mother identifies herself as Hispanic” [56]. The focus on Latinos may inadvertently reinforce misperceptions that undocumented status is an issue solely among Latin American immigrants and obscures the likely significant impact that it has among immigrant groups from Asia, Africa, Europe, or North America.

Finally, each of the two measurement types captures different elements of undocumented status. Self-reported measures attempt to specifically measure a legal element of undocumented status. Proxy measures constitute an approach that relies on social characteristics related to the position of being undocumented. Ultimately, the existing measures in health research are not capturing the same conceptualization of undocumented status and are not validated to capture either its legal or social elements.

### Including measurement issues in discussion of research ethics

Research with undocumented immigrants requires active and critical engagement with ethics. While an in-depth examination of research ethics is beyond the scope of this review, the findings discussed above can guide ongoing discussions of ethics in immigrant health research. Most ethical considerations related to research of undocumented populations have focused on whether or not to ask participants about their legal status [2, 5]. Indeed, to protect research participants, legal status information should only be collected if it advances relevant scientific knowledge. Regardless of whether or not participants are willing to disclose their status, researchers cannot make a total guarantee that information about their legal status will remain confidential. However, ethical consideration also requires that health research address fundamental causes of disease and, if research is conducted relating to undocumented populations, it be done in a rigorous manner that accurately informs policy and practice [17, 57]. The process for measuring undocumented status identified in this review—from data source to information for classifying participants—provides considerations that researchers can assess at each stage of research with undocumented populations.

Researchers who collect legal status data should first weigh the risks and benefits of collecting any sort of data on undocumented status. This should include how researchers will communicate the potential risks to participant, the risks of disclosure, and the plans for how to respond to such disclosures. These considerations, however, should also extend to consideration of the specific risks and benefits of using either direct or proxy approaches in any given study. If it is critical to have data on legal status, researchers should examine ethical considerations of different measurement methods. For example, what ethical considerations should be applied when using proxy measures? Is it ethical to use a person’s characteristic as an approximation of their legal status if the study is entirely anonymous and there is limited disclosure risk? Is it ethical to include an explicit question about undocumented status, as opposed to use of deductive legal status questions, in a longitudinal study that will link data with extensive personal identifiers (e.g., name, phone number, address)? Researchers should be attentive to the unique ethical concerns for each study. The potential risks to privacy and confidentiality and the weighing of risks and benefits will vary based on the specific population under study, the type of measure used, and the context in which the research is conducted. By balancing these concerns and working closely with their Institutional Review Board, researchers can conduct ethically and methodologically sound research.

## Conclusions

Attention to both methodological and ethical issues will, ultimately, improve knowledge on the well-being of undocumented populations. Our findings point to recommendations that can improve the rigor of measurement of undocumented status in health research and indicate areas for further methodological development to fully capture the complexity of undocumented status in a way that is ethically responsive to the experiences of undocumented immigrants.

### Collect self-reported measures of legal status and undocumented status whenever possible and limit the use of proxy measures

When possible, the researcher should ask research participants a set of deductive survey questions regarding their legal status, ending with the explicit question: “Do you have legal authorization to be in the United States?” Given the feasibility of asking about both legal status and undocumented status, in particular, any primary data collection with immigrant populations should integrate such a self-reported measure. Research methodology should take into account the strategy that will be used to develop rapport prior to introducing these questions. This could include the order of the question in a survey or building in additional time to allow participants to decide whether or not to respond. This should also include a survey collection strategy that is sensitive to the research population, such as having co-ethnic researchers conduct the survey, allowing participants to complete their own surveys, or use of telephone or computer surveys.

The use of proxies—as a last resort—should include identification of, rationale for, and discussion of the limitations of the social elements that are serving as approximations of undocumented status. Further, a proxy measure should be avoided entirely if it risks reinforcing stereotypes or misperceptions about undocumented immigrants or erroneously classifying significant numbers of non-undocumented individuals.

### Strengthen the validity of existing measures to create standardized self-reported and proxy measures

The use of validated and standard measures across research studies will allow researchers to compare the experiences of immigrants across populations, geographies, and other personal and contextual factors. Validated and standardized approaches should be developed for self-reported measures in qualitative and survey research by testing and comparing different terms and language that are commonly used to describe legal status: “no papers,” “unauthorized,” or “undocumented.” Qualitative research can be used to assess how different populations of undocumented immigrants identify and speak about legal status in different languages, thus identifying wording that is both accessible and comfortable. Qualitative and quantitative approaches can be triangulated to assess the validity of measures.

Proxy measures can be standardized by establishing standards for the use of common measures, specifically SSN and health insurance data. This should include criteria for when proxies are appropriate (e.g., studies related to health care or insurance) and inappropriate (e.g., conducting population-level estimates).

### Provide detailed methodological information about the measurement process to allow other researchers to assess or replicate measurement approaches

Reporting of measurement methods, including the steps taken to protect research participants, will inform and improve transparency among researchers, as well as provide information about the comparability of findings across studies. Journal editors and peer reviewers can encourage this by asking authors to include this information in their submissions. Authors should specify the type of measure used and details of the information used to classify undocumented participants, such as the wording of survey questions or the steps used to derive a proxy measure, and steps that were taken (e.g., anonymous data collection or a CoC) to protect research participants.

### Develop measures to capture the complexity of the experience of undocumented status for individuals and communities

Undocumented status is not static; therefore, measures should also be developed to capture the elements of individuals’ legal status history, such as changes in status and specific pathways of gaining or losing a status. This should also include measures of other statuses, such as the Deferred Action for Childhood Arrivals [58, 59]. At the family and community levels, the growing recognition of the impact of parent’s legal status on children’s well-being highlights the importance of measuring undocumented status beyond the individual level, such as measures of whether or not a child has an undocumented parent or for mixed-status families [60]. Finally, at the broader contextual level, measures can be developed to capture the structural forces that shape the significance of undocumented status, such as social attitudes or immigration policies.

### Include individuals who are undocumented in the development of measurement methodologies

An implicit assumption in the existing body of research is that immigrant participants do not want to directly discuss issues related to legal status [1, 5]. To avoid making research decisions on assumptions about this populations’ vulnerability and perceptions towards research, this assumption should be tested [3]. As researchers continue to measure undocumented status and build knowledge about the well-being of this population, undocumented immigrants should be engaged in the development and implementation of measures. Researchers can seek input as they grapple with methodological and ethical issues. For example, the validity and reliability of self-reported measures can be strengthened through pilot testing and input on survey development.

Future research in this area can also expand upon the limitations of this systematic review. First, we did not include articles about immigrants in other countries due to the different legal and social systems that produce undocumented statuses. However, similar measurement challenges exist in those contexts. Our recommendations apply across country contexts, but further research on the measurement of both the legal and social elements of undocumented statuses in other countries will provide critical information to health researchers across the globe. In addition, we limited this review to the current health research to obtain an assessment of the state of the field; however, health research would benefit from examining how undocumented status is currently measured in other social science disciplines that use similar data sources and data collection methods.

The increasingly hostile political and social climates towards immigrants in the USA and other regions likely pose significant risks to immigrant health [16]. Rigorous research on the most vulnerable immigrant groups, such as the undocumented, is critical to understanding well-being among immigrant populations. The growing body of research on undocumented status and health has contributed to our understanding of the legal, social, political, and economic significance of this social position. In the future, however, research on immigrant health will require consideration and measurement of undocumented status for a complete understanding of the well-being of these populations. Research on immigrant populations that does not collect this information misses a critical element that affects the access to resources, the sense of security, and rights of these communities. Rigorous methodology is critical for the field to be able to understand and promote the well-being of undocumented immigrants. Our findings provide a starting point for methodological discussions among immigrant health researchers to ensure that individuals with undocumented status are not left in the research shadows.
